# Comment on ‘Cognitive Sarcopenia: Prevalence and the Risk for Mortality and Healthy Aging in the KORA‐Age Study’ by Huemer et al.

**DOI:** 10.1002/jcsm.70286

**Published:** 2026-04-09

**Authors:** Hélio José Coelho‐Júnior, Emanuele Marzetti

**Affiliations:** ^1^ Fondazione Policlinico Universitario Agostino Gemelli IRCCS Rome Italy; ^2^ Department of Geriatrics, Orthopedics and Rheumatology Università Cattolica Del Sacro Cuore Rome Italy

Dear Editor,

1

We read with interest the work by Huemer et al. [1] proposing *cognitive sarcopenia* as a distinct condition defined by the coexistence of sarcopenia and cognitive impairment and associated with an increased risk of adverse outcomes in older adults. While we appreciate the authors' efforts, we believe that both the conceptual foundation and the empirical support for this proposal are insufficient and warrant critical reconsideration.

Our primary concern is that the proposed construct is conceptually derivative of *cognitive frailty*. The original cognitive frailty framework was explicitly intended to extend frailty beyond the physical domain, reinforcing its multidimensional nature and promoting a holistic approach to aging rather than the creation of a new nosological entity [2]. Several years after its introduction, members of the original working group clarified that cognitive frailty was not meant to define a distinct diagnosis and explicitly cautioned against linking cognition and frailty primarily through measurement tools rather than a clearly defined biological substrate [3]. They further warned that the introduction of new terminology had generated an artificial enthusiasm that risked obscuring critical appraisal of both the conceptual framework and its operational limitations [3].

The proposal of cognitive sarcopenia represents an even more problematic extension of this trajectory. Unlike frailty, sarcopenia is intended to describe a specific neuromuscular condition rather than a global state reflecting multisystem decline [4]. Introducing a cognitive dimension into sarcopenia risks conflating a targeted biological construct with domains already routinely assessed within comprehensive geriatric assessment (CGA), thereby offering little conceptual or clinical added value. This concern is amplified by the fact that existing sarcopenia definitions have been repeatedly criticized for their limited evidentiary basis and for reliance on expert opinion rather than robust biological validation [5, 6]. The introduction of yet another construct under these circumstances risks further fragmenting the field and encouraging the proliferation of heterogeneous, noncomparable assessment tools.

From a clinical and epidemiological perspective, the rationale for defining cognitive sarcopenia is also weak. It is well‐established that the coexistence of two age‐related conditions confers a higher risk of adverse outcomes than the presence of either condition alone [3]. Consequently, the observation of increased mortality among individuals meeting criteria for both sarcopenia and cognitive impairment is expected and does not per se justify the introduction of a new entity. As if that alone were not sufficient, the results presented by Huemer et al. [1] do not consistently demonstrate the superiority or added predictive value of the proposed paradigm. Indeed, several methodological limitations substantially undermine the strength of the authors' conclusions.

First, cognitive impairment was assessed using a telephone‐based screening instrument rather than a comprehensive neuropsychological evaluation, limiting diagnostic validity. Second, the article lacks a clear description of participant characteristics, exclusion criteria and the prevalence of frailty, disability, dementia or other conditions known to influence mortality risk. The authors' acknowledgment that relatives and caregivers contributed to data collection further suggests heterogeneity in participants' health status that is insufficiently accounted for. Third, muscle mass was estimated using bioelectrical impedance analysis without adequate control for fasting status or fluid imbalance, despite well‐documented limitations of this method [7, 8]. Fourth, the instrument used to assess disability does not adequately capture the construct of activities of daily living (ADL) [9] and is not comparable to validated tools such as the Barthel Index or Katz Scale [10, 11], as it includes tasks (e.g., running, getting in and out of a car, household management) that extend beyond standard ADL definitions.

Fifth, the prevalence of major confounders, including neurological disease, arthritis, polypharmacy and physical inactivity, was substantially higher in the cognitive sarcopenia group, yet no stratified or sensitivity analyses were performed to disentangle their independent contributions to outcomes. Sixth, although cognitive probable sarcopenia was associated with all‐cause mortality, isolated cognitive impairment also showed statistically significant associations. As no direct comparisons were made between combined and isolated conditions, any inference regarding the superiority of the combined construct remains unsupported. Furthermore, the association for cognitive probable sarcopenia is characterized by limited precision, with confidence intervals approaching the threshold for non‐significance and, in several instances, substantially wider than those observed for isolated conditions. This degree of imprecision suggests inadequate effective sample size and/or marked heterogeneity within subgroups, thereby weakening the stability and interpretability of the effect estimates. In this context, statistically significant point estimates cannot be taken as evidence of a clear or clinically meaningful advantage of the combined construct, particularly given the extensive overlap of confidence intervals across conditions. Seventh, cognitive probable sarcopenia was not consistently associated with adverse outcomes, and in some instances, outcomes typically related to sarcopenia progression (e.g., disability and nursing care) were more strongly associated with isolated conditions than with the combined construct. Finally, the absence of any association between muscle mass and cognition and the consequent failure to examine *confirmed* cognitive sarcopenia alone substantially weakens the rationale for introducing this construct.

In conclusion, while investigating the interaction between sarcopenia and cognitive impairment is clinically relevant, the current evidence does not support the introduction of cognitive sarcopenia as a distinct clinical entity. As previously observed with cognitive frailty, prematurely formalizing a new construct without a clearly defined biological substrate, methodological robustness or demonstrable clinical added value risks generating confusion rather than advancing the field. Future research would be better served by strengthening existing frameworks, improving methodological rigour and integrating multidimensional assessments within established approaches such as CGA, rather than proliferating new and insufficiently substantiated nosological categories.

## Conflicts of Interest

The authors declare no conflicts of interest.

## Data Availability

Data sharing not applicable to this article as no datasets were generated or analysed during the current study.
